# Identification of JNK-JUN-NCOA axis as a therapeutic target for macrophage ferroptosis in chronic apical periodontitis

**DOI:** 10.7150/ijms.102741

**Published:** 2025-01-01

**Authors:** Yuting Wang, Wenlan Li, Wenli Mu, Abdelrahman Seyam, Yonghui Guan, Yifei Tang, Mingfei Wang, Ying Xin, Xiaomei Guo, Tiezhou Hou, Xiaoyue Guan

**Affiliations:** 1 Key Laboratory of Shaanxi Province for Craniofacial Precision Medicine Research, College of Stomatology, Xi'an Jiaotong University, Xi'an, Shaanxi, China.; 2 Clinical Research Center of Shaanxi Province for Dental and Maxillofacial Diseases, College of Stomatology, Xian Jiaotong University, Xi'an, Shaanxi, China.; 3 Department of Cariology and Endodontics, College of Stomatology, Xi'an Jiaotong University, Xi'an, Shaanxi, China.; 4 Department of Urology, The First Affiliated Hospital of Xinjiang Medical University, Urumqi, China.

**Keywords:** JNK-JUN-NCOA axis, macrophages ferroptosis, apical periodontitis

## Abstract

**Objectives:** This study aimed to investigate the involvement of macrophage ferroptosis in chronic apical periodontitis (CAP) and determine if blocking JNK/JUN/NCOA4 axis could alleviate CAP by regulating macrophage ferroptosis.

**Materials and Methods:** Firstly, the *in vitro* models of apical periodontitis (AP) and *in vivo* models of CAP, including clinical specimens and rats' periapical lesions, were utilized to investigate the role of macrophage ferroptosis in CAP by detecting the ferroptosis related factors. The activation of the JNK/JUN/NCOA4 axis was observed in CAP *in vivo* models. Pearson's correlation and linear tendency tests were employed to analyze the correlation between the JNK/JUN/NCOA4 axis and macrophage ferroptosis during CAP progression. Subsequently, the JNK/JUN/NCOA4 axis was blocked by SP600125, and the alterations in ferroptosis associated variables and inflammation levels in macrophages were evaluated.

**Results:** The *in vitro* AP model demonstrated that macrophage ferroptosis mainly occurred during the late phase of inflammatory conditions, with the reduction of GPX4, SLC7A11 and the increase of TFR1 in macrophages. Additionally, a higher accumulation of iron was observed in the periapical lesions derived from clinic samples and animal model. Furthermore, we found that differences in macrophage ferroptosis levels within periapical lesions corresponded altered activation of JNK/JUN/NCOA4 axis. Significantly, the inhibition of JNK/JUN/NCOA4 axis reduced the aforementioned changes and inflammation levels induced by* E. coli* LPS in macrophages.

**Conclusions:** The occurrence of ferroptosis in macrophages contributes to the development of CAP. Targeting the JNK/JUN/NCOA4 axis is an effective therapeutic strategy to rescue the periapical lesions from inflammation due to its anti-macrophage ferroptosis function. Consequently, the current study provides support for further investigation on the JNK/JUN/NCOA4 axis as a targeted signaling pathway for CAP treatment.

## Introduction

Chronic apical periodontitis (CAP) is an inflammatory reaction that occurs when endodontic microorganisms, primarily Gram-negative microbes, invade the root canal system and cause damage to the tissues surrounding the root apex, including periodontal ligament, cementum and alveolar bone^1^. The advancement of CAP frequently coincides with intense pain, swelling discomfort, the enlargement of periapical lesions, or the need for tooth extraction, in addition to its impact on oral health, CAP has also been shown to aggravate the systemic disorders, including metabolic disorders or cardiovascular diseases, all of which considerably diminish patients' life quality^2^, ^3^. Notably, the global incidence of CAP in adults is approximately 6.3%, and it is consistently on the rise^4^, ^5^. Despite standardized treatment approaches including root canal treatment (RCT) and endodontic surgery aim to diminish the microbe infections and provide a favorable healing environment for periapical tissues. The success rate of treatment for CAP is only slightly above 50% after a two-year follow-up, even when performed by experienced endodontic specialists^4^, ^5^. Hence given the predicament in CAP treatment, it is imperative to improve our comprehension of CAP pathology and explore a novel treatment modality for CAP management.

Ferroptosis is a newly identified type of regulated cell death (RCD) that occurs when there is the accumulation of iron-dependent lipid peroxides inside cells^6^. Ferroptosis is distinct from other types of RCD such as apoptosis and cellular pyroptosis^7^. The condition is distinguished by the accumulation of reactive oxygen species (ROS) in lipids and the presence of oxidative stress in cells, which is induced by iron^8^. This leads to cell death but without the typical morphological, biochemical and genetic features seen in other RCDs^7^, ^8^. The main molecular hallmarks of ferroptosis include mitochondrial contraction, the accumulation of ROS, impaired glutathione peroxidase 4 (GPX4), depletion of the glutathione (GSH) and the accumulation of lipid peroxide^9^. More recently, ferroptosis has gained greater recognition as a greater contributor to the development of several physiological and pathological processes, such as infection, inflammation and tumor^9^, ^10^. Importantly, the significance of ferroptosis in oral diseases has been emphasized, particularly in periodontitis and apical periodontitis. Tang *et al.* proposed that ferroptosis plays a catalytic role in the progression of periodontitis by promoting the formation of osteoclasts through the release of inflammatory cytokines, resulting in the worsening of periodontitis^11^. Moreover, Yang *et al.* further investigated the effect of ferroptosis in the progression of apical periodontitis (AP). They discovered that an excessive occurrence of ferroptosis might contribute to bone loss in the periapical region. However, they found that by targeting ferroptosis with ferrostation-1, the development of AP could be greatly inhibited^12^. Macrophages, being the crucial immune cells responsible for maintaining a stable internal environment, serve as the initial barrier of the immune system against invading pathogens causing inflammatory diseases. Macrophages ferroptosis has been linked to a variety of inflammatory diseases, including spinal cord injury, sepsis and atherosclerosis and *et al.*^13^-^15^. Furthermore, macrophages serve as a crucial component in the CAP progression. The above findings strongly suggest that treatments aimed at inhibiting macrophage ferroptosis could be promising strategies in the treatment of CAP.

Nuclear receptor coactive 4 (NCOA4) was first identified as a component of the ret fused gene by Santoro *et al.* in 1994^16^. It has recently been recognized as a ferritin-specific receptor capable of controlling ferritinophage. Research has shown that blocking NCOA4 effectively reduces macrophage ferroptosis in several pathological conditions, including spinal cord injury, septic acute respiratory distress syndrome, chronic obstructive pulmonary disease and *et al.*^13^, ^17^, ^18^. Furthermore, NCOA4 has been shown to be involved in the pathogenesis of oral diseases. Zhang *et al.* found that NCOA4 exhibits significantly higher expression levels in chronic periodontitis samples compared to normal gingival samples^19^. Additionally, Guo *et al.* postulated that NCOA4 contributes to the advancement of periodontitis by exacerbating ROS accumulation and inflammatory responses^20^. Moreover, it has been suggested that manipulating NCOA4 can influence the outcome of several diseases via modulating ferroptosis. Li *et al.* discovered that the suppression of the JNK signaling pathway effectively inhibits the progression of periodontitis by reducing NCOA4. Besides, it has been revealed that the JNK-JUN axis exacerbates osteoarthritis (OA) by regulating NCOA4-mediated ferroptosis and inhibiting the JNK-JUN-NCOA4 axis can mitigate OA development in mice^21^. Given the foregoing, we suggest that targeting JNK-JUN-NCOA4 axis may alleviate CAP by suppressing macrophage ferroptosis.

Taken above, the aim of present study was to investigate the role of macrophage ferroptosis in CAP progression. This was accomplished by examining macrophage ferroptosis in *in vitro* and *in vivo* models. The *in vivo* models not only utilize clinic samples, including healthy periapical tissues, periapical granuloma and periapical cyst, as well as utilizing *in vivo* animal model. Furthermore, the study specifically focused on exploring the activation of JNK/JUN/NCOA4 axis on macrophage ferroptosis in CAP, which was achieved in *in vivo* models including clinical samples and rat AP models. Additionally, the JNK inhibitor, SP600125, was used to further detect whether targeting the JNK-JUN-NCOA4 axis could alleviate CAP by regulating macrophage ferroptosis. Consequently, providing a novel adjuvant therapy agent for the treatment of CAP.

## Materials and Methods

### Clinic samples collection

The present study was approved by the Medical Ethics Committee of Xi'an Jiaotong university Stomatological Hospital (2024-XJKQIEC-QT-0031-001), Xi'an, China. The study followed the declaration of Helsinki and ARRIVE guidelines (https://arriveguidelines.org). The participants in this study were between the ages of 22-50, free of systemic diseases and had not used antibiotics within the previous 6 months. Prior to collecting the clinic samples, the individuals were given detailed information about the study's objectives, and formal informed consent was signed. The inclusion criteria for periapical cyst (RCs) and periapical granuloma (PGs) were consistent with prior investigations^22^. The periapical lesions were diagnosed based on clinical symptoms, X-ray imaging, and histopathological findings as previously described^23^. RCs were defined as lesions in the periapical region of impacted teeth, the presence of fluid or semisolid content observed during endodontic surgery, or the histologic presence of a stratified nonkeratinizing squamous epithelium that totally or partially lines the oral cavity. The PGs were composed of granulomatous tissue including a significant infiltration of inflammatory cells, including lymphocytes, neutrophils, macrophages, *et al.*, however, no epithelial cells were observed. A total of 11 RCs and 10 PGs were identified by dentists and then collected during endodontic surgery. The sample size was determined based on a power calculation from a prior study utilizing G*power version 3.1.97 (https://stats.idre.ucla.edu/other/gpower/, UCLA, USA)^24^. The sample size comprised 31 clinic samples (10 in healthy control group, 11 in periapical cyst group and 10 in periapical granuloma groups) in order to achieve a p-value < 0.05 with 95% power^25^. Healthy periapical tissue samples were obtained from ten subjects who were undergoing orthodontic treatment for permanent tooth extraction. The gathered tissues were promptly preserved in 4% paraformaldehyde for 24h before being embedded in paraffin. The samples were then sectioned into 5μm for further studies.

### Establishment of a rat model for apical periodontitis

The laboratory animal Ethics Committee of the School of Medicine, Xi'an Jiaotong University, approved the animal experiments conducted in the current research (XJTUAE2024-2051). The animal studies were carried out following the AARIVE guidelines and were performed in compliance with the U.K. Animals (Scientific Procedures) Act, 1986 and associated guidelines, EU Directive 2010/63/EU for animal experiments. Before the operation, the rats were acclimated for a week. Then, a total of forty male Sprague Dawley (SD) rats, aged 6-8 weeks and weighing between 180-220g, were randomly assigned into 5 groups based on the experimental design: 0d, 7d, 14d, 21d and 28d, with eight rats per group. The animal sample size was confirmed according to the previous study using G*power version 3.1.97 (https://stats.idre.ucla.edu/other/gpower/, UCLA, USA)^24^. The results revealed that 8 rats each group can achieve a p-value < 0.05 with 95% power^25^. Apical periodontitis was induced in each group of rats by exposing the dental pulp of bilateral mandibular first molars (**Fig. 3B**). Concisely the rats were anesthetized (intraperitoneal injection) by ketamine (70mg/kg boy weight; Sigma-Aldrich, USA) and xylazine (6mg/kg body weight, Sigma-Aldrich, USA). Next, a 1/4# small round ball drill was employed to drill into the bilateral mandibular first molar, 10K# endodontic files were used to locate the root canals, and the dental pulp was exposed to oral environment. Notably, according to the previous study, the invasive endodontic cavity was located on the distal-occlusa surface^26^. At the specified time, the bilateral mandibles were collected and fixed with 4% paraformaldehyde for 36h at room temperature. The left side of the mandibles were subjected to Micro-CT and histology analysis. The right mandibles were embedded in paraffin and sectioned longitudinally at 4μm thickness in mesio-distal orientation then used for further immune testing.

### Micro-CT

The fixed left hemimandibles were scanned using a cone beam-type tomograph (QuantumGX, PerkinElmer, Hopkinton, MA, USA). The scanning parameters were configured as follows: the voltage was set to 70kV, the current to 114mA, the FOV to 25mm, and the voxel size to 50μm. The films that were scanned were subsequently analyzed using dimension image analysis software Mimics 17.0 (Materialize, Leuven, Belgium) in a double-blinded manner. Following Micro-CT analysis, the obtained samples underwent histology analysis.

### Hematoxylin-Eosin (HE) staining

The paraffin slices were dewaxed by incubating with xylene I for 8min for a duration of 8min, followed by incubation with xylene II for another 8min. Next, the specimens were rehydrated using a series of alcohol gradients, including 100% ethanol, 95% alcohol, 85% alcohol and 75% alcohol, with each step lasting 3min. Then washed with distilled water. After that, the sections were immersed in hematoxylin for 20min, followed by two washes with tap water, and finally stained with eosin for a period of 2min. Following the process of dehydration, stained sections were examined using a light microscope (Olympus, Tokyo, Japan). The images were captured at original magnifications of ×20 and ×40.

### Prussian blue reaction

In order to investigate the presence of iron-containing heme in periapical region, periapical tissue sections were subjected to staining using Prussian blue iron stain kit (Solarbio, China). The staining process involved treating the samples with Perls staining buffer for 30min, as instructed by the manufacture. Subsequently, the samples were washed with distilled water for 50s and lightly stained with eosin staining solution for 30s to enhance the background. After a brief rinse with distilled water for 3s, the samples were dehydrated using anhydrous ethanol. The presence of iron-containing heme was observed and documented using a light microscope (Olympus, Tokyo, Japan).

### Immunohistochemical (IHC) staining

Immunohistochemistry for IL-1β was conducted using the method of streptavidin-Biontin Complex (SABC) method, following the manufacturer's protocol from Boster, China. The slices pre-treatment similar to the one described in HE staining. Next, the sections were incubated with digestive solution (Boster, China) to retrieve the antigens at a temperature of 37℃ for a duration of 30min. Subsequently, the samples were treated with 3% hydrogen peroxide to eliminate the endogenous peroxidase. The samples were then treated with 5% BSA to prevent non-specific binding and then exposed to the appropriate primary antibody anti-IL-1β (1:100 dilution, Santa Cruz, CA, USA). After 20 h, the slides underwent treatment with secondary antibody (Zhongshanjinqiao, Beijing, China) for a period of 1.5 h. The immune response was detecting using the DAB substrate kit (Boster, Wuhan, China). The analysis was performed using the Image J software (NIH, Bethesda, MD, USA) to provide semi-quantitative results.

### Immunofluorescence (IF) double staining

For IF double staining, periapical tissues and the right mandibles of rats that had been fixed with paraformaldehyde were subjected to dehydration, embedding, and slicing using the steps as those described in HE staining. Next, the slides were immersed in 3% H_2_O_2_ solution to retrieve endogenous peroxidase. Subsequently, the samples were blocked with 5% BSA, followed by incubation of the tissue's samples with primary antibodies against CD68&GPX4, CD68&SLC7A11, CD68&TFR1, CD68&NCOA4, CD68&P-JNK and CD68&JUN. Notably, the dilution of the primary antibodies mentioned above was 1:150, the primary antibody CD68 was purchased from Bioss, China and the others were purchased from Santa Cruz, USA. Next, the secondary antibodies (diluted at a ratio of 1:50, Boster, Wuhan, China) used were goat anti-rabbit CY3 or goat anti-mouse FITC. The nucleus was counterstained with DAPI. The immunology-positive cells were observed and record using a confocal microscope (Olympus, Japan). Semi-quantitative analysis was conducted using Image J software (NIH, Bethesda, MD, USA).

### Cell culture and treatments

RAW264.7 macrophages were purchased from Procell Life Science & Technology Co., Ltd. (Wuhan, China). The macrophages were grown in a Dulbecco's modified Eagle's medium (DMEM, Gibico, USA) with the addition of 10% bovine serum (Procell Life Science & Technology Co., Ltd. Wuhan, China). The cells were then placed in a humidified incubator set at a temperature of 37 ℃ and a CO2 concentration of 5%. Once the cells attained a confluence of 70%, a concentration of 100ng/ml *E. coli* LPS was added to the cells at various time intervals (0, 0.5, 1, 6, 12 and 24h) to imitate the acute or chronic inflammatory conditions in the infected periapical region^27^, ^28^.

The JNK inhibitor SP600125 (Sigma, USA) was utilized to assess the impact of JNK-JUN-NCOA4 axis on macrophage ferroptosis. Initially, the macrophages underwent pretreatment with SP600125 (10μM) for 1h^27^. Afterwards, the medium was removed, and the cells were washed twice with PBS (Boster, China). Then the cells were subjected to further treatment with 100ng/ml *E. coli* LPS for 24h.

### Western-blot analysis

The Macrophages were lysed in RIPA buffer supplemented with 1mM phenylmethanesulfonyl fluoride. The supernatant was collected after spinning the samples at a centrifuge of 12000g for 15min at 4℃. The protein concentrations were determined using a bicinchoninic acid (BCA) kit (Boster, China). Equal amounts of protein (30μg) were mixed with loading buffer (Boster, China) at a 4:1 ratio. Next, the protein samples were segregated using sodium dodecyl sufate polyacrylamide gel. Subsequently, the proteins were transferred onto polyvinylidene fluoride (PVDF) membranes (Millipore, USA). The membranes were then blocked with 5% fat-free milk or 5% BSA solution for 1.5h. Afterwards the membranes were subjected to incubation with several primary antibodies, including GPX4 (1:1000, Bioss, Beijing, China), TFR1 (1:1000, Bioss, Beijing, China), SLC7A11 (1:1000, Bioss, Beijing, China), P-JNK (1:500, Santa Cruz, USA), JNK (1:500, Santa Cruz, USA), C-JUN (1:500, Santa Cruz, USA), NCOA4 (1:500, Santa Cruz, USA), overnight at 4 ℃. Following three washes in TBST for a duration of 10min each, the membranes were incubated with the secondary antibody in a dilution of 1:3000. The immunological blots were observed using ECL detection reagent (Millipore, USA). GAPDH was designated as the internal controls for the total protein. The densitometry of the bands from western blot experiment was analyzed using Image J software (Version 8, NIH, Bethesda, MD, USA).

### Quantitative RT-PCR

The total RNA was obtained using the TRIzol reagent procedure (Thermo Fisher Scientific, Waltham, MA, USA). The purity and concentration of the collected RNA were assessed using a NanoDrop (Thermo Scientific, USA). Subsequently, the RevertAid First Strand cDNA synthesis kit (Thermo Fisher Scientific, MA, USA) was employed following the guidelines provided by the manufacturer. A SYBR-green based reverse transcription polymerase chain reaction (RT-PCR) was conducted using 50 amplification cycles. The temperature conditions for each cycle were as follows: 94°C for 1 minute, 56°C for 1 minute, and 72°C for 2 minutes. The RT-PCR was carried out using an ABI PRISM 7700 device manufactured by Applied Biosystems, based in Woburn, Massachusetts, USA. The mRNA levels of these targets were standardized based on the expression of GAPDH, and the relative expression levels of the target genes were measured using the 2-△△CT method. The precise primers utilized in this study are displayed in **Table 1.**

### Measurement of intracellular reactive oxygen species (ROS)

Raw264.7 macrophages were distributed evenly in 24 well plates, with a density of 20×10^3^ cells per well. The cells were subjected to different stimuli based on their assigned experimental groups. The levels of ROS were evaluated using Reactive oxygen species assay kit (Beyotime, China) following the instructions provided by the manufacture. The DCFH-DA was mixed with serum-free medium in a ratio of 1:1,000 to get a final concentration of 10 μM/L. The cells were then exposed to a pre-diluted DCFH-DA probe at a temperature of 37°C. After 20 min, the cells were rinsed with serum-free medium three times in order to eliminate any surplus DCFH-DA probe that did not penetrate the cells. Thermo Scientific™ Varioskan™ LUX multimode microplate reader was employed to detect the fluorescence intensity using an excitation wavelength of 488 nm and an emission wavelength of 525 nm.

### Measurements of Malonaldehyde (MDA), Glutathione (GSH) and Superoxide dismutase (SOD)

To investigate the presence of macrophage ferroptosis, we purchased GSH and GSSG Assay Kit, Lipid Peroxidation MDA Assay Kit and Total Superoxide Dismutase Assay kit with Wst-8, all from Beyotime in China. The studies were carried out in accordance with the instructions provided by the manufacture. Concisely, macrophages were seeded in 6-well plates with a density of 1×10^5^ cells/ml. The cells were exposed to various stimuli according to their designated experimental groups. The cells were obtained through trypsin digestion and then subjected to sonication in an ice bath. Next, the macrophages underwent processing in accordance with the appropriate detection kit. The microplate reader (Thermo Scientific™ Varioskan™ LUX, USA) measured absorbances at wavelengths of 532 nm, 412 nm, and 450 nm.

### Network pharmacology analysis

Firstly, the identification of CAP-associated disease genes was performed by searching two databases, the DisGeNet (https://www.disgenet.org/, accessed on 15 January 2023) and GeneCards (https://www.genecards.org/, accessed on 15 January 2023) using the term “chronic apical periodontitis” as the search query. The ultimate set of genes related with CAP was generated by eliminating any duplicate entries that were detected. Next, the ferroptosis target genes included in CAP were subjected to the KEGG enrichment analyses employing the Metascape database (https://www. metascape.org, accessed on 15 Janurary 2023). The Metascape database is a web-based portal that offers comprehensive gene list annotation and analysis resources for experimental biologists^29^. The criteria for screening in KEGG enrichment analyses were set as follow: Min overlap = 3 and Min Enrichment = 1.5. A The P-value < 0.01 was deemed significant. Subsequently, we explored the correlation between P-JNK, NCOA4, ferroptosis and CAP by utilizing the DisGeNet (https://www.disgenet.org/, accessed on 15 January 2023) and GeneCards (https://www.genecards.org/, accessed on 15 January 2023) databases. The common targets were visualized using the Venny software (Verision number: 2.1.0). After that, the STRING database (https://cn.string-db.org/, accessed on 15 January 2023) was used to draw the PPI network with the objective of identifying the shared genes among the aforementioned parameters.

### Transmission electron microscopy (TEM) assays

The use of TEM was employed as the preferable approach to examine ferroptosis by observing the ultrastructure of mitochondrion^30^. The experimental procedures were conducted in accordance with previously stated methods^31^. In brief, the cells were cultured on a 6-well plate at a density of 1×10^5^ cells per well. The cells were exposed to different stimuli according to their allocated experimental groups then subsequently collected using trypsin digestion. Next, the cells were rinsed with PBS three times, followed by fixation with 2.5% glutaraldehyde for 6h. Subsequently, the samples were dehyhrated using a gradient of alcohol and finally embedded in resin. The observation was conducted using TEM (JEM1400FLASH, Japan).

### Statistical analysis

Statistical analysis was performed using SPSS software (SPSS Inc., Chicago, IL, USA). The graphic software utilized was GraphPad Prism 8.0 (GraphPad Software, Inc., La Jolla, CA, USA). All data are presented as mean ± SD. A one-way analysis of variance (ANOVA) was used for various group comparisons. The Pearson's correlation and linear tendency tests were used to examine the relationship between linked immunological components. A two-tail P-value < 0.05 was considered statistically significant; *R^2^* > 0.8 indicates a high correlation; 0.5 ≤ R^2^ ≤ 0.8 indicates a moderate correlation; 0.3 ≤ *R^2^* ≤ 0.5 indicates a poor correlation; and *R^2^* < 0.3 indicates no correlation. The studies were performed in duplicate and repeated a minimum of three times.

## Results

### Changes in macrophage ferroptosis were observed *in vitro* under inflammatory conditions

**Figure 1:**
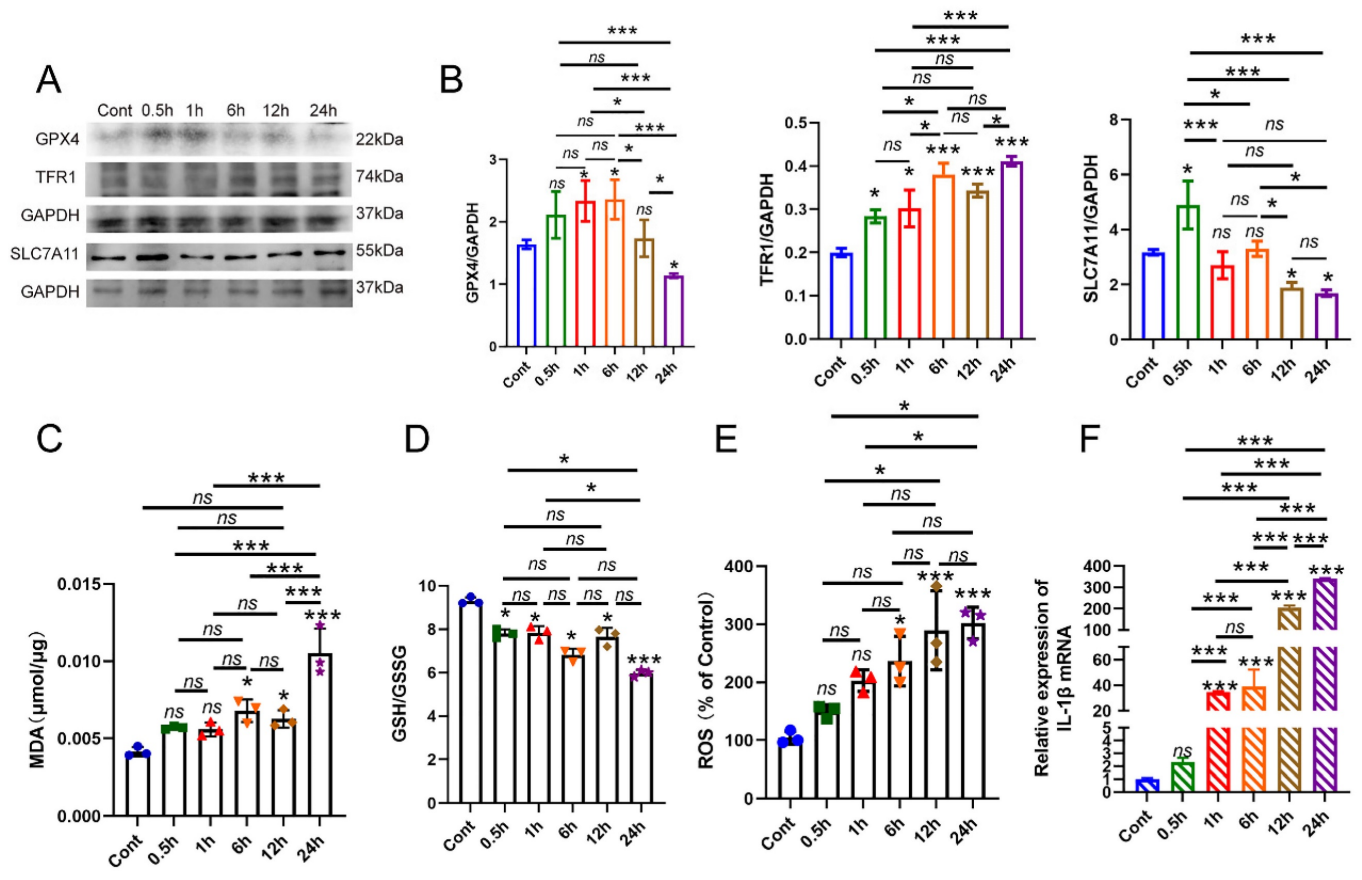
The involvement of macrophage ferroptosis in the advancement of apical periodontitis. (A, B) The AP laboratory model was established by subjecting macrophages to 100ng/ml *E. coli* LPS at time points: 0, 0.5, 1, 6, 12 and 24h. Among them, 0h was set as control. The representative images and quantification of GPX4, TFR1, SLC7A11 in western-blot analysis. (C, D) The expression of GSH, MDA in each group in macrophages. (E) The identification of ROS accumulation in each group. (F) Real-time PCR results of IL-1β in each group. Data were presented as mean ± SD. *ns P* > 0.05, *P < 0.05, ***P < 0.01.

In the previous study, we successfully established a laboratory model of apical periodontitis by subjecting Raw 264.7 macrophages to a concentration of 100ng/ml *E. coli* LPS. Thus, we firstly identify the presence of macrophage ferroptosis *in vitro* model. The macrophages were exposed to *E. coli* LPS for various durations: 0.5h, 1h, 6h, 12h and 24h. The macrophages that did not receive *E. coli* LPS treatment were designated as the control group (Cont). Firstly, RT-PCR was conducted to confirm the inflammatory status of *E. coli* LPS treated groups. The results showed that the treatment of *E. coli* LPS significantly increased the expression of IL-1β mRNA in each group, and the expression of IL-1β in 24h group arrived the peak value (**Fig. 1F, *P* < 0.05**). Western-blot analysis was recruited to evaluate the protein levels of GPX4, SLC7A11 and TFR1, which are markers of ferroptosis. Initially, the protein level of GPX4 exhibited a sightly increase at each time point after being treated with *E. coli* LPS for 1h, 6h (**Fig. 1A, B, *P* < 0.05**). Then, the protein concentration of GPX4 exhibited a decline following 12h and 24h of *E. coli* LPS treatment. Nevertheless, there was no notable distinction found between the Cont group and 12h group (**Fig. 1A, B, *P* > 0.05**). SLC7A11 expression was also detected. Compared to the Cont group, the expression of SLC7A11 showed a considerable increase in the 0.5h group, followed by a quick drop in the 1h, 6h, 12h and 24h groups. No discernible distinction was observed in group 1h and 6h. Furthermore, we additionally identify the presence of TFR1 expression in each group. The administration of *E. coli* LPS significantly raised the expression of TFR1 in each group (**Fig. 1A, B, *P* < 0.05**).

Furthermore, the levels of MDA, GSH, and ROS were also measured. The findings demonstrated that the levels of MDA were marginally higher after 6h of treatment with *E. coli* LPS and markedly increased after 12h of treatment with *E. coli* LPS compared to the Cont group (**Fig. 1C, *P* < 0.05**). Subsequently, we conducted a thorough analysis of the GSH levels in each group and observed a substantial drop in the GSH levels in the *E. coli* LPS groups, particularly following a 24h treatment with *E. coli* LPS (**Fig. 1D, *P* < 0.05**). Moreover, the levels of ROS were also identified. The findings demonstrated a significant rise in ROS levels following treatment with *E. coli* LPS for 6h, 12h and 24h treatment with *E. coli* LPS (**Fig. 1E, *P* < 0.05**). Based on the aforementioned data, it is suggested that the ferroptosis in macrophages primarily occurs during the late and chronic stages of inflammation.

### Macrophage ferroptosis is involved in the pathogenesis of apical periodontitis

**Figure 2:**
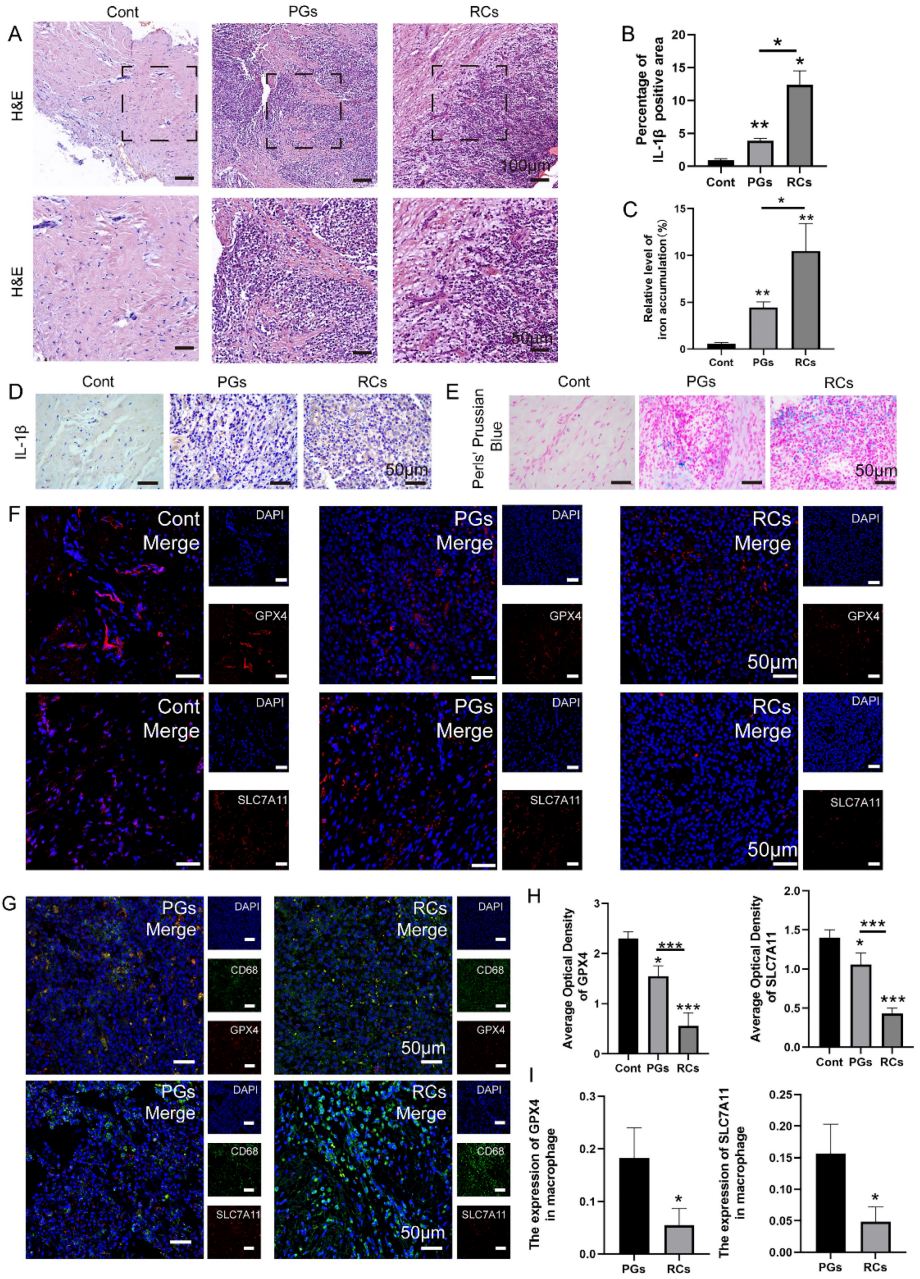
The content of macrophage ferroptosis was increased in chronic inflamed periapical lesions. (A) Representative images of H&E staining of Cont and CAP groups (including PGs and RCs). Scale bar: the upper=200μm, the lower=50μm. (B, D) Representative images and the quantification of IL-1β positive staining cells in Cont, PGs and RCs groups. Scale bar=50μm. (C, E) The iron content was detected by Prussian blue staining. Representative images and the quantification of iron content in Cont, PGs and RCs groups. Scale bar=50μm. (F, H) Representative images and the quantification of GPX4 and SLC7A11 positive staining cells in Cont, PGs and RCs groups. Scale bar=50μm. (G) GPX4-CD68 and SLC7A11-CD68 double labeling in PGs and RCs groups. (I) The ratio of the GPX4, SLC7A11 positive macrophages in total macrophages were quantified in PGs and RCs groups. Data were presented as mean ± SD. *P < 0.05, ***P < 0.01, ns. *P*>0.05. Cont, healthy control; PGs, periapical granulomas; RCs, radicular cysts.

To investigate the involvement of macrophage ferroptosis in CAP, we collected clinic samples of healthy periapical tissues (Cont) and chronically inflamed periapical tissues harboring periapical cysts (RCs) and periapical granulomas (PGs). The inflammatory status of periapical tissues was assessed by conducting HE staining on tissue slices (**Fig. 2A**). The findings revealed that the periapical lesions contain a substantial quantity of inflammatory cells, including lymphocytes, neutrophils and macrophages, in contrast to the healthy periapical tissues that are predominantly composed of fibroblasts (**Fig. 2A**). Furthermore, IHC staining demonstrated a higher level of IL-1β expression in periapical lesions. The results revealed that the expression of IL-1β in periapical lesions is greater than that in healthy periapical tissue, particularly in RCs (**Fig. 2B, D, *P* < 0.05**). About the presence of ferroptosis, we firstly detect the expression of GPX4 and SLC7A11 in periapical sections of clinical tissues. We discovered a notable reduction in the expression of GPX4 and SLC7A11 in inflamed periapical regions, particularly in RCs group, in comparison to the healthy periapical tissue (**Fig. 2F, H, *P* < 0.05**). Next, we performed an additional investigation on macrophage infiltration in the periapical tissue region. In healthy periapical tissues, there is rare macrophage infiltration can be observed (**Fig. 2A**). However, within the inflamed periapical tissue, particularly in RCs, there was a noteworthy rise in the quantity of macrophage (CD68^+^) (**Fig. 2G, I, *P* < 0.05**). In addition, we detect the expression of GPX4 and SLC7A11, which are indicators of ferroptosis, in macrophages located in periapical lesions using via IF double-staining. The findings demonstrated the expression of GPX4 and SLC7A11 in the infiltrating macrophages (CD68^+^) of periapical lesions (**Fig. 2G, I**). The results showed that compared to PGs group, the expression of GPX4 and SLC7A11 in RCs group decreased significantly (**Fig. 2F, H,* P* < 0.05**). Furthermore, the findings demonstrated that as the co-localization of CD68^+^&GPX4 and CD68^+^&SLC7A11 decreased in the periapical region, the expression of IL-1β was up-regulated correspondingly (**Fig. 2G, I, *P* < 0.05**).

To further corroborate the occurrence of macrophage ferroptosis in apical periodontitis, the collected periapical tissues were subjected to prussian blue staining, the results indicated that the iron deposition in periapical lesions was stronger than that in healthy periapical region (**Fig. 2C, E,* P* < 0.05**). Moreover, a stronger amount of iron deposition was observed in RCs compared to PGs (**Fig. 2C, E, *P* < 0.05**). The data above suggests that macrophage ferroptosis plays a role in the development of chronic apical periodontitis, and the increased occurrence of macrophage ferroptosis may contribute to the exacerbation of chronic apical periodontitis.

### Progression of CAP in animal model was in correlation with the occurrence of macrophage ferroptosis

**Figure 3:**
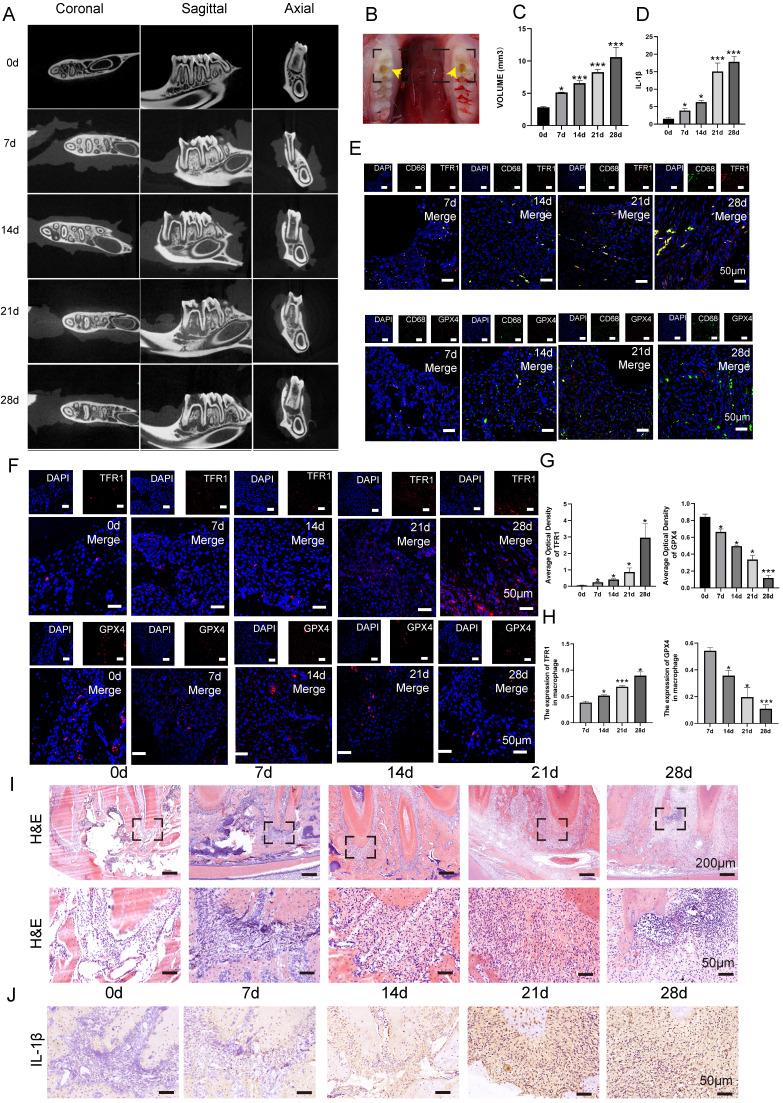
The content of macrophage ferroptosis during the development of chronic apical periodontitis animal model. (A, B, C) The establishment of experimental CAP model. Representative Micro-ct 3-dimentiaonal reconstruction images of mandible first molar of CAP at different stages as indicated (A). Image of pulp exposure-induced chronic apical periodontitis model (B). Quantification of bone resorption volumes at different development times of CAP. The obvious bone resorption emerged on day 7 and peaked on day 28 (C). (E) TRF1-CD68 and GPX4-CD68 double labeling in the periapical lesions in the 7, 14, 21 and 28d after pulp exposure. (F, G) Representative images (F) and the quantification of TRF1 and GPX4 positive staining cells (G) in different groups. Scale bar=50μm. (I) Representative images of H&E staining of each group. Scale bar: the upper=200μm, the lower=50μm. (J, D) Representative images (J) and the quantification of IL-1β positive staining cells (D) in different groups. Scale bar=50μm. (H) The ratio of the TRF1 and GPX4 positive macrophages in total macrophages were quantified in the periapical lesions in the 7, 14, 21 and 28d after pulp exposure. Data were presented as mean ± SD. *P < 0.05, ***P < 0.01, ns. *P* > 0.05.

In order to investigate the role of macrophage ferroptosis in chronic apical periodontitis, we establish a CAP model in forty SD rats (**Fig. 3B**). Following pulp exposure, the rats were euthanized in a sequential manner on days 0, 7, 14, 21 and 28, with 8 rats in each group. Micro-ct and HE demonstrated that the volume of bone resorption in periapical regions progressively increased over time (**Fig. 3A, C, I, *P* < 0.05**). Among above, the mandibles that were collected on day 0 after the pulp was exposed were designated as the control group. On day 0, the periapical tissues surrounding the root foramen were undamaged and few inflammatory cells were present (**Fig. 3C, I, *P* < 0.05**). Furthermore, there was no evidence of bone resorption (**Fig. 3A, C, *P* < 0.05**). As apical periodontitis progresses, the alveolar bone undergoes resorption, which serves as a sign of CAP. In detail, alveolar bone loss in periapical lesions was found 7 days after pulp exposure (**Fig. 3A, C, *P* < 0.05**). Subsequently, the bone loss extended in the horizontal, coronal, and sagittal planes from day 7 to 28 (**Fig. 3A, C,* P* < 0.05**). Furthermore, an increasing number of inflammatory cells were detected as rats' CAP advanced (**Fig. 3I**). In addition, we further conducted additional research on the expression of the pro-inflammatory cytokine IL-1β in periapical lesions. As anticipated, the protein level of IL-1β showed a substantial increase from 0 to day 28 (**Fig. 3J, D, *P* < 0.05**).

To study the involvement of macrophage ferroptosis in CAP, we proceeded to analyze the protein levels of TFR1 and GPX4, which are known markers of ferroptosis, in periapical tissues during the rats' CAP progression. **Fig. 3F** demonstrated a high number of GPX4 positive cells in the day 0 group. However, as CAP advanced, there was a considerable drop in the expression of GPX4. Unlike GPX4, the presence of TFR1 immuno-positive cells is infrequent on day 0 after pulp exposure. However, when CAP develops, the expression of TFR1 significantly increases and reaches its highest level on day 28 after pulp exposure (**Fig. 3F, G, *P* < 0.05**). Next, we proceed to identify the expression of GPX4 and TFR1 in infiltrating macrophages. The IF co-staining analysis revealed a reduction in the expression of GPX4 and an increase in the expression of TFR1 in infiltrating macrophages (CD68^+^) (**Fig. 3E, H, *P* < 0.05**). Our findings indicate that as the size of lesion volumes increased during the course of inflammation, the proportion of GPX4 positive macrophages to total macrophages in each group decreased accordingly (**Fig. 3E, H, *P* < 0.05**). Whereas, the portion of TFR1 positive macrophages to total macrophages in each group increased correspondingly (**Fig. 3E, H, *P* < 0.05**). Taken together, the aforementioned studies indicate that macrophage ferroptosis may aggravating the advancement of CAP.

### The results of Network pharmacology analysis

**Figure 4:**
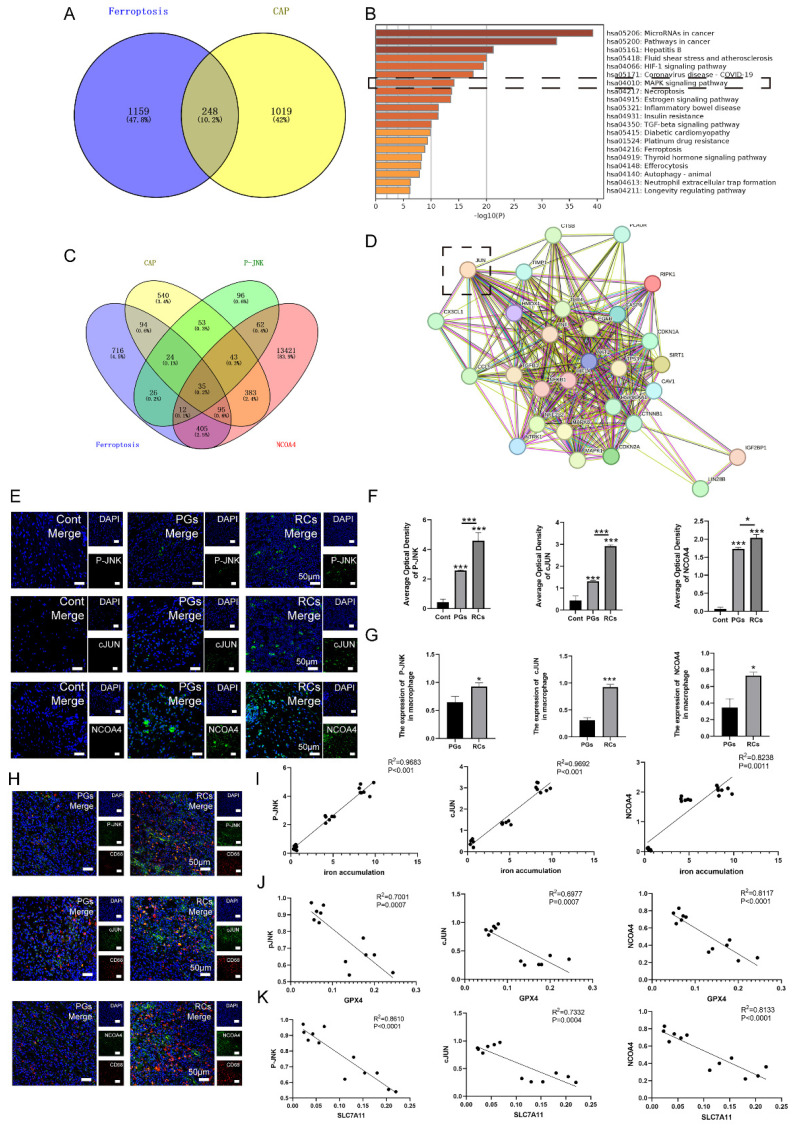
The correlation between JNK/JUN/NCOA4 axis and macrophage ferroptosis in the clinic samples. (A) Venn diagram of 248 common targets from CAP and ferroptosis. (B) KEGG pathway analysis of ferroptosis in treating CAP. (C) Venn diagram of 35 common targets from CAP, ferroptosis, JNK and NCOA. (D) PPI network analyzed the connecting genes between JNK and NCOA4. (E, F) The representative images (E) and the quantification of P-JNK, c-JUN and NCOA4 (F) in Cont, PGs and RCs groups. Scale bar=50μm. (H) P-JNK-CD68, c-JUN-CD68 and NCOA4-CD68 double labeling in the PGs and RCs groups. Scale bar=50μm. (G) The ratio of the P-JNK, c-JUN and NCOA4 positive macrophages in total macrophages were quantified in the PGs and RCs groups. (I, J, K) Pearson's correlation and linear tendency analysis revealed the positive correlation between JNK/JUN/NCOA4 axis and the expression of GPX4(J), SLC7A11(K) and the iron accumulation(I) (all of the above parameters were presented in Figure 2) in PGs and RCs groups. Data were presented as mean ± SD. *P < 0.05, ***P < 0.01, ns. *P* > 0.05. *R^2^* > 0.8 indicates a high correlation; 0.5 ≤ *R^2^* ≤ 0.8 indicates a moderate correlation; 0.3 ≤ *R^2^* ≤ 0.5 indicates a poor correlation; and *R^2^* < 0.3 indicates no correlation.

Initially, we investigated whether JNK/JUN/NCOA4 axis could mitigate CAP via modulating macrophage ferroptosis by network pharmacology analysis. The results interpreted that a total of 248 intersecting genes were identified between the targets of ferroptosis and CAP, which were considered prospective biological targets for ferroptosis in CAP treatment (**Fig. 4A**). Moreover, the KEGG pathway enrichment analysis identified the pathways associated with ferroptosis that are engaged in CAP therapy, including MAPK signaling pathway (**Fig. 4B**). In order to further detect the role of JNK/JUN/NCOA4 axis in ferroptosis during CAP treatment, we examined the relationship between P-JNK, NCOA4, ferroptosis and CAP. The findings indicated that there are 35 genes that are common shared among the parameters mentioned above (**Fig. 4C**). Furthermore, the protein interaction network diagram elucidated that JUN plays a pivotal role in connecting JNK and NCOA4 (**Fig. 4D**). To summarize, the above results indicate that targeting JNK/JUN/NCOA4 axis could potentially influence the progression of CAP by modulating ferroptosis.

### The expression of JNK/JUN/NCOA4 axis varies depending on CAP development *in vivo* setting

To verify the role of JNK/JUN/NCOA4 axis in regulating macrophage ferroptosis in CAP progression, we analyzed the expression of JNK/JUN/NCOA4 axis in macrophages using both human periapical samples and rats' periapical tissue samples. Firstly, analysis of IF labeling in human clinic samples revealed a significant upregulation of P-JNK, JUN, and NCOA4 expression located in the periapical region, when compared to healthy periapical tissue (**Fig. 4E, F, *P* < 0.05**). Furthermore, the RCs group exhibited higher levels of P-JNK, JUN and NCOA4 positive macrophages (CD68^+^) compared to PGs group (**Fig. 4G, H, *P* < 0.05**).

Besides, the findings discovered a positive correlation between the heightened activation of JNK/JUN/NCOA4 axis and the increased expression of IL-1β in the periapical region (**Fig. 4E, F, Fig. 2D**). In addition, we have identified the correlation between JNK/JUN/NCOA4 axis and GPX4, SLC7A11. The results demonstrated a negative correlation between P-JNK&GPX4 (*R^2^* = 0.7001, *P* < 0.001), JUN&GPX4 (*R^2^* = 0.6977, *P* < 0.001), NCOA4&GPX4 (*R^2^* = 0.8117, *P* < 0.001), P-JNK&SLC7A11 (*R^2^* = 0.8610, *P* < 0.001), JUN&SLC7A11 (*R^2^* = 0.7332, *P* < 0.001), NCOA4&SLC7A11 (*R^2^* = 0.8133, *P* < 0.001, **Fig. 4J, K**). Additionally, a positive correlation was identified between P-JNK&iron deposition (*R^2^* = 0.9683, *P* < 0.001), JUN&iron deposition (*R^2^* = 0.9692,* P* < 0.001), as well as NCOA4&iron deposition (*R^2^* = 0.8238, *P* < 0.05, **Fig. 4I**). The above suggested that the JNK/JUN/ NCOA4 axis may involve in the development of human CAP by regulating macrophage ferroptosis.

**Figure 5:**
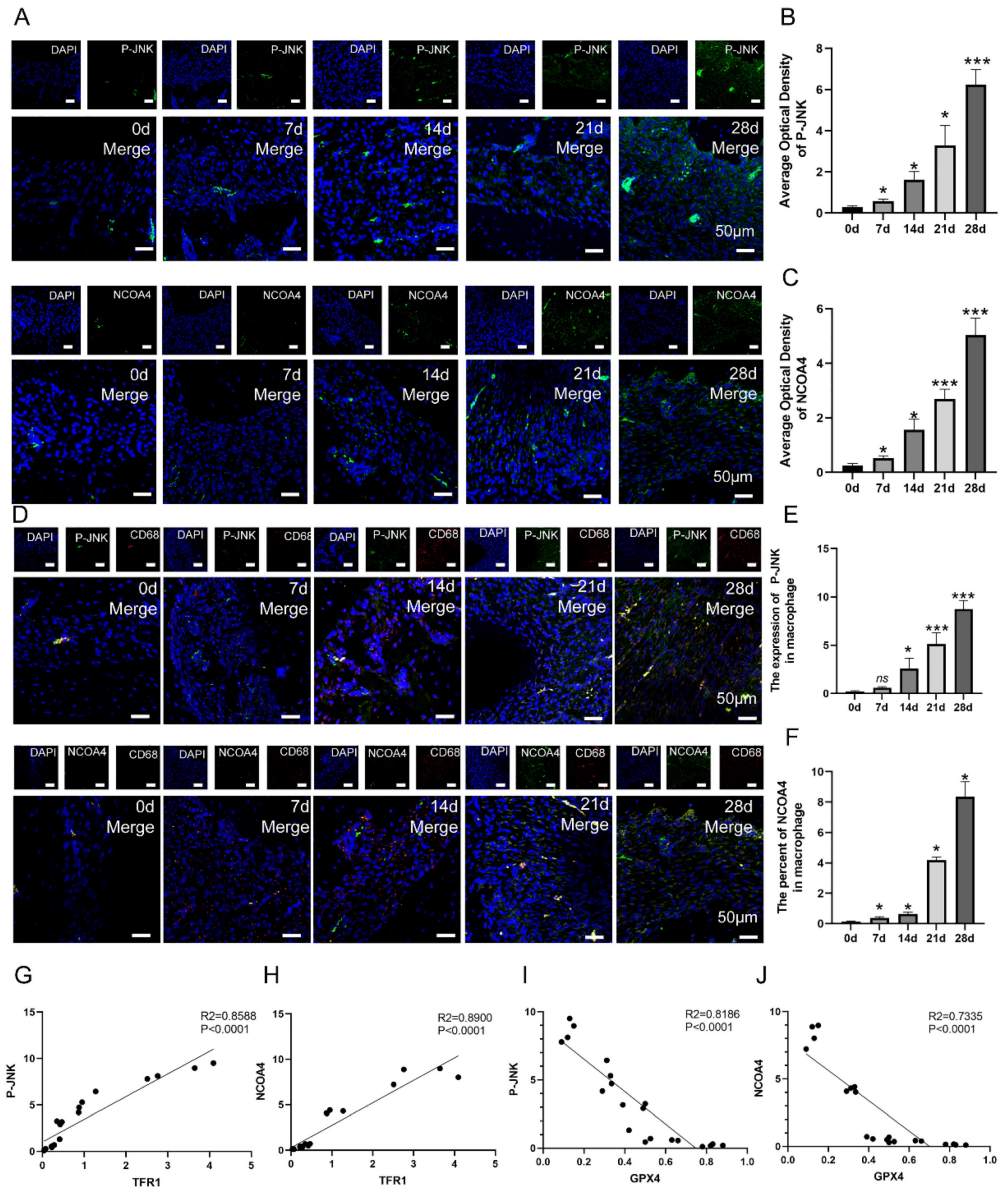
The correlation between JNK/JUN/NCOA4 axis and macrophage ferroptosis in the development of CAP in rat model. (A, B, C) The representative images (A) and the quantification of P-JNK(B) and NCOA4(C) positive staining cells in the periapical lesions in each group. Scale bar=50μm. (D, E, F) The representative images (D) and the quantification of P-JNK-CD68(E) and NCOA4-CD68(F) double labeling in total macrophages in periapical lesions in the 7, 14, 21 and 28d after pulp exposure. Scale bar=50μm. (G, H, I, J) Pearson's correlation and linear tendency analysis revealed the positive correlation between P-JNK&TFR1(G), P-JNK&GPX4(I), NCOA4&TFR1(H), NCOA&GPX4(J) (the expression of TFR1 and GPX4 in the development of CAP were presented in Figure 3) in each group. Data were presented as mean ± SD. *P < 0.05, ***P < 0.01, ns. *P*>0.05. *R^2^* > 0.8 indicates a high correlation; 0.5 ≤ *R^2^* ≤ 0.8 indicates a moderate correlation; 0.3 ≤ *R^2^*≤ 0.5 indicates a poor correlation; and r < 0.3 indicates no correlation.

To verify whether JNK/JUN/ NCOA4 axis participates the progression of CAP in animal model via regulating macrophage ferroptosis, we further detect the expression of P-JNK, NCOA4 in macrophages in periapical lesions. The expression of P-JNK and NCOA4 in periapical regions showed an increase from day 0 to day 28 after pulp exposure (**Fig. 5A-C, *P* < 0.05**). Besides, the number of P-JNK and NCOA4 positive macrophages (CD68^+^) in periapical regions showed an increase from day 7 to day 28 after pulp exposure (**Fig. 5D-F, *P* < 0.05**). Next, we further analyze the correlation between P-JNK, NCOA4 and macrophage ferroptosis using Pearson's correlation and linear tendency analysis. The findings indicated a negative correlation between P-JNK&GPX4 (*R^2^* = 0.8186, *P* < 0.001, **Fig. 5I**), as well as between NCOA4&GPX4 (*R^2^* = 0.7335, *P* < 0.001, **Fig. 5J**). Nevertheless, a robust positive correlation was found between P-JNK&TFR1 (*R^2^* = 0.8588, *P* < 0.001, **Fig. 5G**), as well as NCOA4&TFR1 (*R^2^* = 0.8900, *P* < 0.001, **Fig. 5H**). These results provide additional evidence that the JNK/JUN/NCOA4 axis plays a significant role in regulating macrophage ferroptosis during CAP progression.

### Inhibiting JNK/JUN/NCOA4 axis by SP600125 effectively suppress macrophage ferroptosis in an *in vitro* model of CAP

**Figure 6:**
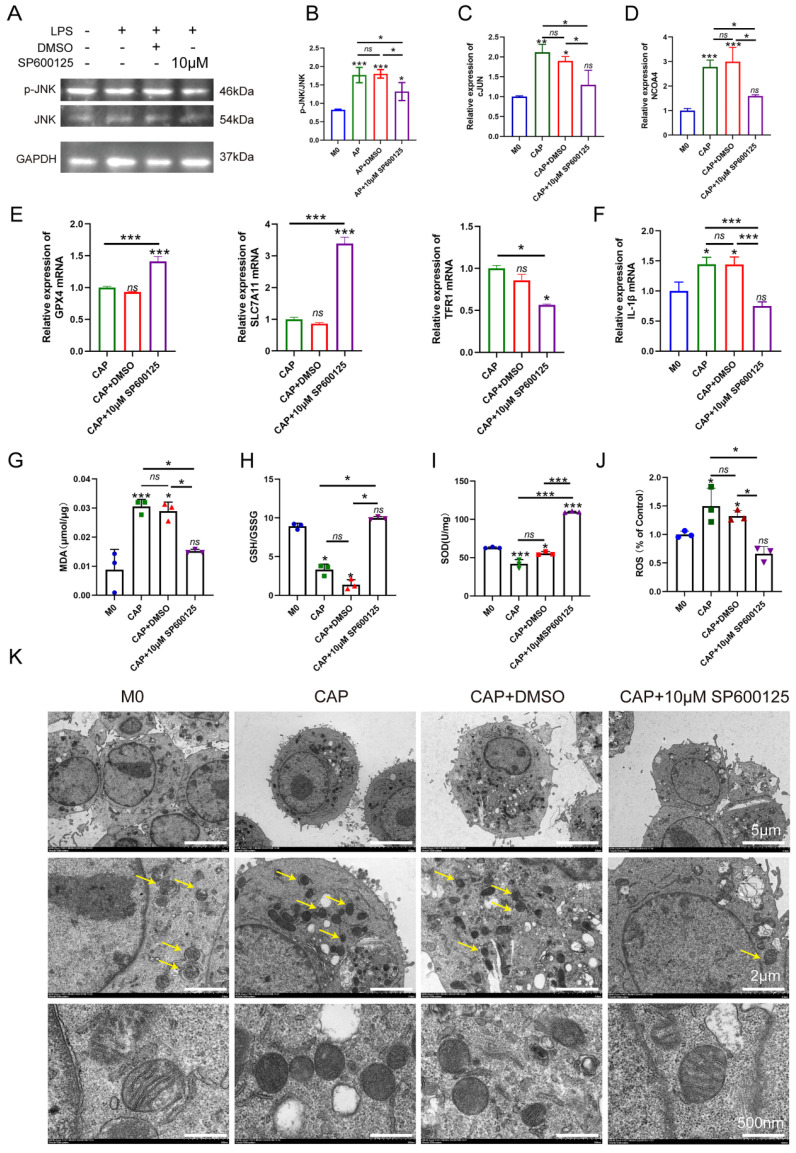
Inhibition of JNK/JUN/NCOA4 axis by SP600125 in CAP in *E. coli* LPS induced inflammatory response suppressed the content of macrophage ferroptosis and the inflammatory status. (A, B) The representative images(A) and quantification of P-JNK and JNK(B) in western-blot analysis. (C, D) Real-time PCR results of c-JUN(C) and NCOA4(D) in each group. (E) Real-time PCR results of GPX4, SLC7A11 and TFR1 in each group. (F) Real-time PCR results of IL-1β in each group (G-I) The expression of MDA(G) and GSH(H), SOD(I) in each group in macrophages. (J) The detection of ROS accumulation in each group. (K) the representative TEM images of mitochondria in each group. Scale bar: the upper=5μm, the middle=2μm, the lower=50μm. Data were presented as mean ± SD. *P < 0.05, ***P < 0.01.

To further confirm the role of JNK/JUN/NCOA4 axis in regulating macrophages during CAP, we conducted an experiment in *E. coli* LPS-induced inflammatory response. Raw264.7 macrophages were treated with 100ng/ml *E. coli* LPS for 24h, while the JNK signaling pathway was blocked using a specific inhibitor, SP600125 (Sigma, USA). Subsequently, we investigate the effects of SP600125 on JUN, NCOA4 expression and the extent of macrophage ferroptosis. SP600125 demonstrated significant inhibition of JNK phosphorylation, as well as the expression of C-JUN and NCOA4 caused by *E. coli* LPS treatment, when compared to untreated controls (**Fig. 6A-D, *P* < 0.05**). Importantly, we further found that when exposed to *E. coli* LPS-induced inflammatory condition, inhibiting the activity of the JNK/JUN/NCOA4 axis with SP600125 significantly enhances the expression of GPX4, SLC7A11 when compared with those in CAP and CAP+DMSO groups (**Fig. 6E, *P* < 0.05**). Furthermore, markedly reduces the expression of TFR1 (**Fig. 6E, *P* < 0.05**). Notably, no significant difference in the aforementioned indicators was observed between the CAP and CAP+DMSO groups. In addition, in comparison with CAP and CAP+DMSO groups, the application of SP600125 significantly reduce the accumulation of ROS and MDA levels. Besides, the levels of GSH and SOD have been greatly restored (**Fig. 6G-J, *P* < 0.05**). Furthermore, the morphological features of macrophage mitochondria were assessed through transmission electron microscopy to provide further evidence of the involvement of JNK/JUN/NCOA4 axis in macrophage ferroptosis (**Fig. 6K**). As depicted in **Fig. 6K**, compared with the control group, the mitochondrial volume in macrophages from the CAP and CAP+DMSO treatment group exhibited a reduction in mitochondrial volume and an increase in mitochondrial membrane density, as compared to the control group (**Fig. 6K**). Particularly, SP600125 could block the aforementioned *E. coli* LPS induced changes (**Fig. 6K**). Yellow arrows in **Fig. 6K** indicate the morphological characteristics of mitochondrial in the different groups. In addition, we also detect the expression of IL-1β in the presence of SP600125 during an inflammatory condition and uncovered that the application of SP600125 significantly decrease the expression of IL-1β (**Fig. 6F, *P* < 0.05**). Thus, we have demonstrated that the blockade of JNK/JUN/ NCOA4 axis could reduce the severity of macrophage ferroptosis and thereby lessen the CAP severity.

## Discussion

Recent evidence has demonstrated the role of macrophage ferroptosis in exacerbating inflammation progression, particularly in periodontitis and apical periodontitis^12^, ^20^, ^31^. In current study, we initially unveiled that macrophage ferroptosis participates in the pathology of chronic apical periodontitis and contributes to its progression. Moreover, since the JNK signaling pathway has been associated with the regulation of macrophages' biological function and the JNK/JUN/NCOA4 axis has been found to regulate the ferroptosis of various cell types, we conducted further research to determine the role of the JNK/JUN/NCOA4 axis in modulating the chronic apical periodontitis course by regulating macrophage ferroptosis. Through the establishment of the *in vitro* and *in vivo* models, we have discovered that there is a direct correlation between the JNK/JUN/NCOA4 axis and the presence of macrophage ferroptosis during chronic apical periodontitis progression. Furthermore, this study initially verified that blocking JNK/ JUN/NCOA4 axis can effectively suppress macrophages ferroptosis, resulting in a decrease in the inflammatory status associated with chronic apical periodontitis.

Macrophages are identified to have a crucial role in the apical periodontitis progression by regulating inflammatory responses in periapical lesions^32^. Ferroptosis is defined as an iron and lipid peroxidation dependent form of programmed cell death^9^. In the previous studies, the investigation of macrophages ferroptosis mainly focus on the chronic inflammatory diseases. Wu *et al.* discovered a heightened incidence of macrophage ferroptosis in lethal sepsis. They also found that enhancing macrophage ferroptosis worsens the lethal sepsis progression, while suppressing it can shield the individuals from physical damage^33^. Xiao *et al.* further confirmed the above findings that suppressing macrophage ferroptosis can reduce the inflammatory response in pulmonary sepsis injury^34^. Moreover, the effect of macrophage ferroptosis on chronic inflammatory diseases has also been demonstrated in systemic sclerosis model. The researcher unveiled that an increase in macrophage ferroptosis could aggravate the progression of fibrosis in systemic sclerosis, while the inhibition of macrophage ferroptosis can alleviates fibrosis during the course of systemic sclerosis^28^. In addition to systemic diseases, ferroptosis has also been linked to oral diseases, such as chronic periodontitis and apical periodontitis. Wang *et al.*, Fu *et al.* and Qiao *et al.* conducted studies using an animal model of periodontitis^31^, ^35^, ^36^. They discovered that ferroptosis plays a role in the development of periodontitis. Additionally, they also demonstrated that targeting ferroptosis could suppress the progression of periodontitis. Furthermore, the involvement of ferroptosis also referred in apical periodontitis. Through the establishing of *in vitro* and *in vivo* AP model, Yang *et al.* unveiled the involvement of ferroptosis in AP and further demonstrated that the suppression of ferroptosis could inhibit the progression of AP^12^. In this study, considering the ease of acquiring the CAP laboratory model and our successful establishment of the CAP *in vitro* model, we firstly investigate the presence of macrophage ferroptosis in the *in vitro* CAP model. Previous researches indicated that stimulating Raw264.7 cells with 100 ng/ml LPS for 24 h can effectively mimic chronic apical periodontitis *in vitro*^32^, ^37^. Furthermore, to investigate the role of ferroptosis in the development of apical periodontitis, we designed 6 timelines to simulate the progression of apical periodontitis. In addition, the timelines of *in vivo* models are confirmed based on the previous researches. The previous study showed that periapical bone defects appeared 7 days after pulp exposure and peaked at 28 days^38^. Consequently, we selected 0d, 7d, 14d, 21d, 28d as the timelines for the *in vivo* models. Based on the above findings, we supposed that the presence of macrophages ferroptosis may account for the chronic inflammatory response in CAP development. During the early phase of apical periodontitis, there is a response increase in the expression of GPX4 and SLC7A11 to protect the AP model from being damaged by ferroptosis. Moreover, regarding the macrophage subtypes in CAP, specifically M1 and M2 macrophages, the researchers have further explored whether macrophage phenotypes are associated with ferroptosis. Cao *et al.* stressed that M1 macrophages could enhance the sensitivity of macrophage ferroptosis under inflammatory condition^28^. Gu *et al.* reported that macrophage ferroptosis promoted the polarization of M1 macrophage^39^. Besides, Wang *et al.* demonstrated that in the pathogenesis of neutrophilic airway inflammation, inhibiting macrophages polarized into M1 subtype could effectively suppress the occurrence of macrophage ferroptosis^40^. These studies indicated that ferroptosis is tightly associated with macrophage M1 polarization. Given that in chronic inflammatory periapical lesions, the infiltration of M1 macrophage significantly exceeds that of the M2 phenotype^41^, we inferred that M1 macrophage infiltration may facilitate the occurrence of macrophage ferroptosis, hence exacerbating the CAP course. Future studies will be conducted to further investigate the relationship between macrophage polarization and macrophage ferroptosis. Taken above, these findings indicate that macrophage ferroptosis may play a key role in the pathology of CAP.

Ferroptosis is strongly affected by cellular oxidative stress^6^. Redox glutathione (GSH) plays a critical role in ferroptosis by serving as the preferred substrate for GPX4, which is essential for avoiding ferroptosis^42^. Our current findings in clinical samples revealed that the inflamed periapical tissues displayed a reduction in the expression of GPX4 when compared with healthy control tissues, especially in RCs group. For GPX4 is essential for cells to carry out ferroptosis, thus we conducted additional analysis on SLC7A11, a particular subunit of the cystine/glutamate antiporter, which functions as a suppressor of ferroptosis by ensuring a stable redox state and also enhances the production and durability of GSH^42^, ^43^. The results indicate that the inflamed periapical tissues exhibit a notable decrease in the protein levels of SLC7A11. Iron is a crucial ingredient for the accumulation of lipid peroxides and the onset of ferroptosis^7^. Previous study elucidated that excessive iron ions have a propensity to generate a significant quantity of ROS through a fenton chemical reaction^44^. Hence, the accumulation of Fe^3+^ in periapical lesions has also been observed in clinic samples, and the results indicate that as CAP progresses, there is a significant rise in iron deposition in RCs. Moreover, to provide additional evidence of the involvement of macrophage ferroptosis in the development of CAP, we conducted further analysis to detect ferroptosis in macrophage (CD68^+^) using co-IF staining. For rarely macrophages can be seen in healthy periapical tissues, we only investigate the co-localization of GPX4&CD68, SLC7A11&CD68 in PGs and RCs. The results unveiled that there was a higher prevalence of macrophage ferroptosis in RCs compared to PGs. To provide additional evidence about the effect of macrophage ferroptosis on the CAP course, we additionally establish CAP animal model to examine the role macrophage ferroptosis by assessing the levels of GPX4 and TFR1 in periapical lesions. TFR1 facilitates the transport of iron ions from extracellular to the intracellular^45^ and the elevated expression of TFR1 has been reported to promote the cellular uptake of a substantial quantity of iron ions, which is responsible for triggering ferroptosis^45^. In accordance with the clinic samples, it was observed that there was a rise in macrophage ferroptosis occurrence when there was an expansion in both the volume of bone loss and the extent of inflammatory status in periapical lesions. Notably, the impact of macrophage ferroptosis in CAP has been clarified in previous study. Yang *et al.* investigate the involvement of macrophage ferroptosis in CAP by using healthy periapical tissues and the inflamed periapical tissues^12^. Whereas, the inflamed periapical tissues were not further classified into granulomas or radicular cysts. Furthermore, the emphasis on the activation of macrophage ferroptosis during the course of CAP has not been addressed yet^12^. Our study initially identified the involvement of macrophage ferroptosis in the development of CAP by analyzing clinic samples, including RCs and PGs. Furthermore, we also examined the participation of macrophage ferroptosis in the CAP progression animal model. Thus, the above findings are highly fascinating and suggest that by suppressing the activation of macrophage ferroptosis, it may be possible to prevent inflammation in periapical lesions and hinder the CAP course.

Prior research has revealed that the JNK signaling pathway plays a crucial role in the development of ferroptosis. Zhao *et al.* showed that the activation of JNK signaling pathway could aggravate the ferroptosis of colorectal cancer cells by inducing NCOA4. Moreover, the colorectal cancer cells' ferroptosis could be effectively reduced by suppressing NCOA4 through the inhibition of the JNK signaling pathway with SP600125^46^. In addition, Sun *et al.* Provided further clarification that the inhibition of the JNK-JUN-NCOA4 axis could alleviate osteoarthritis via decreasing chondrocyte ferroptosis^21^. Consist with previous studies, we discovered a strong association between the expression of P-JNK, c-JUN and NCOA4 in macrophages and the biomarkers of macrophage ferroptosis (GPX4, SLC7A11, and TFR1) in the development of CAP in both clinical samples and animal models. This suggest that JNK-JUN-NCOA4 axis may participate in the progression of CAP via regulating macrophage ferroptosis. In order to confirm the above findings, macrophages were treated with the JNK signaling pathway inhibitor SP600125. As a result, the phosphorylation of JNK, as well as the expression of c-JUN and NCOA4 were, were considerably suppressed. Significantly, the inhibition of JNK-JUN- NCOA4 axis could successfully prevent the macrophage ferroptosis and inflammatory status in CAP cell model. When considering the information presented, these studies confirm that targeting the JNK/c-JUN/NCOA4 axis could ameliorate CAP by suppressing macrophage ferroptosis.

To summarize, the data presented in current study indicate that macrophages ferroptosis is involved in the progression of chronic apical periodontitis. Remarkably, our findings from both *in vitro* and *in vivo* experiments uncovered a novel role of the JNK/JUN/ NCOA4 axis in regulating macrophage ferroptosis in chronic apical periodontitis. This study may provide a novel perspective on a potential therapeutic strategy for chronic apical periodontitis.

## Conclusions

The data presented in current study indicate that macrophages ferroptosis is involved in the progression of chronic apical periodontitis. Remarkably, our findings from both *in vitro* and *in vivo* experiments uncovered a novel role of the JNK/JUN/ NCOA4 axis in regulating macrophage ferroptosis in chronic apical periodontitis. This study may provide a novel perspective on a potential therapeutic strategy for chronic apical periodontitis.

## Figures and Tables

**Table 1:** Primers used in RT-PCR analysis.

Gene	Forward primer (from 5' to 3')	Reverse primer (from 3' to 5')
Gapdh	GGTGAAGGTCGGTGTGAACG	GTGGTAGAAGGTCCTCGCTC
SLC7A11	CTCCGAGGAGCAAGAGGAGTA	ATCACTGTTCGGTCGTGACT
TFR1	TTCGCAGGCCAGTGCTAGG	ACAAGGGAGTACCCCGACAG
GPX4	CAAAGTCCTAGGAAACGCCC	GAGGGGTCATGACGTTGTCG
cJUN	TGGGCACATCACCACTACAC	CCGACTTGACGTATCGGTCT
NCOA4	TGTCTGGGTCGGTCTAAGGT	CTCCACGTCACTACGTTCCT
IL-1β	AGACAACTGCACTACAGGCT	TTCCTCTTGGTTCGTTGCTGTT
